# Correction: Halogen-bonded charge-transfer co-crystal scintillators for high-resolution X-ray imaging

**DOI:** 10.1039/d4sc90091j

**Published:** 2024-05-20

**Authors:** Yu-Hua Chen, Guo-Zhen Zhang, Fu-Hai Chen, Shu-Quan Zhang, Xin Fang, Hong-Ming Chen, Mei-Jin Lin

**Affiliations:** a Key Laboratory of Advanced Carbon-Based Functional Materials (Fujian Province University), College of Chemistry, Fuzhou University Fuzhou P. R. China meijin_lin@fzu.edu.cn fangxin@fzu.edu.cn; b College of Materials Science and Engineering, Fuzhou University Fuzhou P. R. China chm@fzu.edu.cn; c College of Zhicheng, Fuzhou University Fuzhou P. R. China

## Abstract

Correction for ‘Halogen-bonded charge-transfer co-crystal scintillators for high-resolution X-ray imaging’ by Yu-Hua Chen *et al.*, *Chem. Sci.*, 2024, https://doi.org/10.1039/d4sc00735b.

The authors regret that there were errors in ref. 2, 4, 8, 13, 20 and 36 of the original article. The correct references are presented herein as ref. 1–6.

The Royal Society of Chemistry apologises for these errors and any consequent inconvenience to authors and readers.

## Supplementary Material
